# Fluorescence *in-situ* hybridisation on biopsies from clam ileocystoplasties and on a clam cancer

**DOI:** 10.1038/sj.bjc.6603035

**Published:** 2006-03-21

**Authors:** K D Ivil, S H Doak, S A Jenkins, E M Parry, H G Kynaston, J M Parry, T P Stephenson

**Affiliations:** 1 Department of Urology, The University Hospital of Wales, Cardiff, UK; 2 Centre for Molecular Genetics and Toxicology, University of Wales, Swansea, UK; 3 Academic Department of Surgery, Clinical School, University of Wales, Swansea, UK

**Keywords:** aneusomy, cystoplasty, fluorescence *in-situ* hybridisation, genetics, aneuploidy

## Abstract

The incidence of carcinoma following an enterocystoplasty increases with time and is a major concern after such procedures. The aim of this study was to investigate genetic instability (in the form of numerical chromosomal aberrations) at the enterovesical anastomosis in patients who had undergone a clam ileocystoplasty using fluorescent *in-situ* hybridisation (FISH). Fluorescent *in-situ* hybridisation was performed on touch preparation samples prepared from fresh endoscopic biopsies obtained from the enterovesical anastomosis and native bladder remnant (control specimens) of 15 patients who had undergone a clam ileocystoplasty. Fluorescent *in-situ* hybridisation was also performed on one squamous cell cancer specimen. Significant aneusomic changes were found at the enterovesical anastomosis in all 15 patients. Alterations in chromosome 18 copy number were the most frequent abnormal finding (trisomy 18, *n*=8; monosomy 18, *n*=7). Nine patients were monosomic for chromosome 9. Isolated monosomy 8 and trisomy 8 were each found in one patient. The control specimens were all normal. An unusually high incidence of polysomic cells was found in the clam tumour specimen, reflecting the aggressive nature of this cancer. Chromosomal numerical abnormalities occur at the enterovesical anastomosis following a clam ileocystoplasty and chromosome 18 appears to be a particularly good marker of genetic instability. The results of this study indicate that morphologically normal tissue obtained from the enterovesical anastomosis displays evidence of chromosomal instability that may predispose to tumour formation. However, further prospective, blinded, longitudinal studies are required to establish whether predetermined FISH signal patterns in enterocystoplasty cells in urine or obtained by biopsy predict the presence or absence of tumour.

The incidence of carcinoma following an enterocystoplasty increases with time and there is an urgent need to develop techniques to identify patients who are at risk of developing a life-threatening tumour (Ali-El-Dein *et al*, 2002). Fluorescence *in-situ* hybridisation (FISH) of exfoliated bladder cells has been shown to have a greater sensitivity than conventional urine cytology in the detection of transitional cell carcinoma of the bladder (Halling *et al*, 2000). Possibly therefore, detection of early chromosomal changes indicative of genomic instability at the enterovesical anastomosis in patients who have undergone enterocystoplasty may identify those at high risk of tumorigenesis before a potentially fatal cancer has occurred.

Preliminary studies using comparative genomic hybridisation (CGH) identified amplifications on chromosomes 9, 18 and 21 in deoxyribonucleic acid (DNA) extracted from biopsies obtained from the enterovesical anastomosis of patients who had undergone a clam ileocystoplasty. These chromosomal abnormalities were also present in DNA obtained from the tumours arising in augmentation cystoplasties, but not in any control samples obtained from the native bladder remnant (Appanna *et al*, 2000; Appanna, 2004). Amplifications on chromosome 8 were only observed in the augmentation tumours investigated. Although CGH is a useful cytogenetic technique that assesses chromosomal alterations on a global scale, its sensitivity is limited as it relies on bulk tissue analysis. Consequently, rare abnormalities, such as those in a subgroup of cells within premalignant lesions, may be missed. In contrast, interphase FISH is a single-cell analysis-based technique, which allows the detection of vary rare alterations and is therefore more sensitive than CGH. Therefore, the purpose of the present study was to further investigate abnormalities involving chromosomes 8, 9 and 18 (an appropriate FISH probe specific to chromosome 21 alone is not currently commercially available) in patients who had undergone a clam ileocystoplasty using FISH to detect early genetic changes at the enterovesical anastomosis. Material from a ‘clam’ tumour was also investigated using FISH.

## MATERIALS AND METHODS

### Patient population

Touch sample preparations were obtained from 15 patients undergoing rigid cystoscopy and biopsy of the urothelium at the enterovesical anastomosis. In seven patients a clam enterocystoplasty had been performed for the treatment of a neuropathic bladder and in eight for treatment of an overactive non-neuropathic bladder (four of whom had a congenitally unstable bladder). All patients were augmented with ileum. The average age of the patients at the time of their biopsy was 35.9 years (median 31 years), with a range of 21–62 years. The average time from the construction of a clam enterocystoplasty to the present study was 12.5 years (median 12 years), with a range of 6–18 years.

#### Case report

A 53-year-old female presented with painless haematuria. She had spina bifida occulta and had undergone a clam ileocystoplasty at the age of 45 for the treatment of a neuropathic bladder. A cystoscopy diagnosed a tumour of the anterior bladder remnant close to the enterovesical anastomosis. The patient underwent a laparotomy, where it was found that the tumour involved the symphysis pubis. A radical cystectomy, hysterectomy, bilateral oophorectomy and ileal conduit were performed. Despite radiotherapy, the patient died the following year. Macroscopic examination demonstrated a 30 mm × 20 mm ulcerating tumour on the anterior wall of the bladder. The neoplasm was located in a urothelial area extending to within 10 mm of the ileal margin. Light microscopy revealed a moderately differentiated squamous cell carcinoma invading into the perivesical fat. The urothelium adjacent to the tumour displayed histological features characteristic of carcinoma *in situ*, these changes extending as far as, but not crossing the ileovesical anastomosis. Away from the tumour, the bladder and bowel epithelium were inflamed, with no dysplasia. Material taken from the symphysis pubis showed invasive carcinoma in the fibrous tissue, but no involvement of bone.

Part of the tumour was dissected from the cystectomy sample immediately following operation. Touch-sample preparations were prepared from the fresh tumour material and stored at −20°C.

### Collection of biopsy samples

Approval for this work was obtained from the local hospital ethics committee and informed consent was obtained from all patients. It is standard practice in our unit that patients who have undergone a clam enterocystoplasty are followed up yearly by performing rigid cystoscopy and biopsy of the urothelium at the enterovesical anastomosis. In addition to the usual anastomotic biopsy for histology, where possible a biopsy was taken from the enterovesical anastomosis and from the native bladder remnant at least 3 cm from the enterovesical anastomosis (control specimen).

### Touch-sample preparations

Touch-sample preparations were prepared with fresh biopsy material. Bladder biopsies were touched repeatedly onto a microscope slide, taking care not to smear the sample across the slide. The slides were air-dried for 10 min, immersed twice in methanol/acetic acid 3 : 1 (Fisher Scientific, Hampshire, UK) for 20 min each and air dried for 30 min. Successful touch-sample preparations were stored at −20°C.

### Touch-sample pretreatment

Tissue touch preparations were digested with pepsin (300 *μ*g pepsin, 100 ml 0.01 M HCl, pH 2.8) at 37°C for 7 min. The digestion was arrested by immersing slides in phosphate-buffered saline (PBS) solution for 5 min at room temperature, PBS containing 50 *μ*M magnesium chloride at room temperature for a further 5 min and air dried.

### Fluorescence *in-situ* hybridisation

Predigested touch-sample preparations were dehydrated in an ethanol series and air-dried. Orange, green and aqua fluorescent centromeric probes (Vysis, Surrey, UK) were used to identify chromosomes 8, 9 and 18, respectively. All three probes in hybridisation buffer were simultaneously placed on the target area of each slide, co-denatured on a hot plate at 75°C for 2 min and hybridised in a humidified chamber at 37°C for 16 h. The slides were subsequently immersed in 0.4 × SSC/0.3% Nonidet-40 (NP-40) at 73°C for 2 min, 2 × SSC/0.1% NP-40 at room temperature for 30 s and air-dried. The samples were counterstained with 10 *μ*l 4′,6′-diamidino-2-phenylindole hydrochloride (DAPI; Vysis, Surrey, UK) and evaluated under an Olympus BX50 microscope equipped with single and multiple band-pass filters to visualise orange, green and aqua fluorescent probes. Images were captured using Macprobe v4.3 (PowerGene) image analysis software. In all, 200 non-overlapping nuclei with clearly visible boundaries were scored for each sample.

### Statistical significance

Normal values were obtained by analysing the data obtained from the control specimens. The cutoff criteria used in defining results as normal *vs* aberrant were defined as 3s.d.'s from the mean of the control values for each of the chromosomes studied. Consequently, significant monosomy was considered to be present when it occurred in >6% of the cells and significant trisomy when it occurred in >4% of cells. Regression analysis was performed using Microsoft Excel software.

## RESULTS

Fluorescent *in-situ* hybridisation was successfully performed on test samples prepared from all 15 patients (Table 1) and 12 of the 15 control specimens (Table 2). No chromosomal abnormalities were observed in any of the control samples. However, significant aneusomic changes were found at the enterovesical anastomosis of all 15 patients despite there being no dysplastic or malignant changes, observations which suggest that chromosomal abnormalities are present in patients with normal histology.

**Figure 1:**
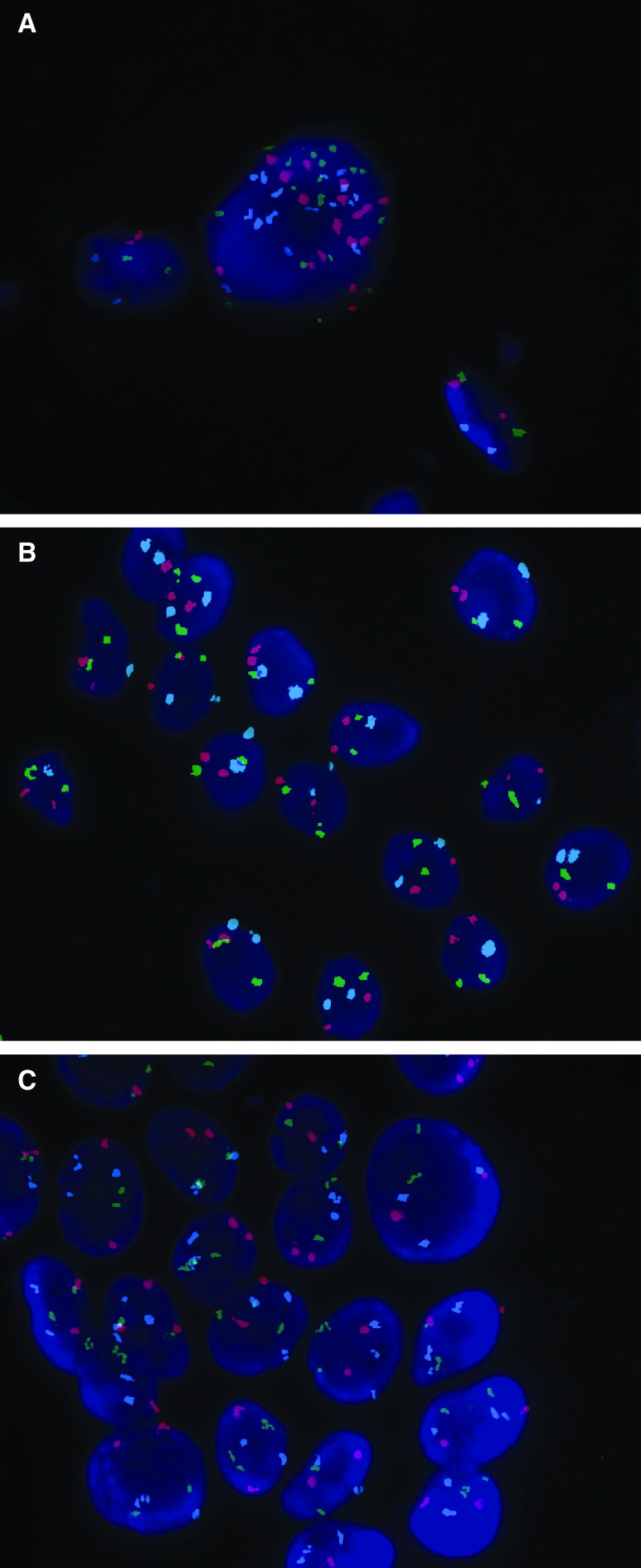
(**A**) Several nuclei from the enterovesical anastomosis demonstrating chromosome 18 monosomy. (**B**) Examples of chromosome 18 trisomy in cells originating from a histologically normal enterovesical anastomosis. (**C**) A highly polysomic cell deposited from a clam tumour (orange – chromosome 8; green – chromosome 9; aqua – chromosome 18).

Alterations in chromosome 18 copy number were the most frequent abnormal finding and were almost evenly divided between monosomy (53%; Figure 1A) and trisomy (47%; Figure 1B). Two patients had both a gain and loss of chromosome 18. Nine patients (60%) were monosomic for chromosome 9, but no cases of trisomy 9 were found. Only two patients had chromosome 8 copy number abnormalities; monosomy 8 and trisomy 8 were each found in one patient. In both these patients, cells with monosomy and trisomy of chromosome 8 abnormalities in copy number of chromosome 18 were also present, suggestive of several chromosomal aberrations. Aneusomy involving at least two chromosomes was observed in nine patients.

Regression analysis indicated that neither the total number of aneusomic cells (*r*^2^=0.008) nor the extent of aneusomy of chromosome 8 (*r*^2^=0.149), chromosome 9 (*r*^2^=0.131) or chromosome 18 (*r*^2^=0.189) was influenced by the latent period between patients undergoing a clam ileocystoplasty and the collection of biopsies. Furthermore, the age of the patient did not affect the number of aneusomic cells detected (*r*^2^=0.127).

Fluorescent *in-situ* hybridisation performed on touch preparations obtained from a clam tumour specimen indicated that these tumours are highly chromosomally unstable. Trisomy of chromosomes 8 and 9 as well as both monosomy and trisomy of chromosome 18 were observed (Table 3), with only 38% of cells containing two signals for all the three chromosomes studied. The most striking genetic feature of the tumour material was the high level (23%) of polysomy (Figure 1C). Furthermore, although the majority of polysomic cells were either trisomic or tetrasomic, some showed as many as 16 signals for a given probe (Figure 1C).

## DISCUSSION

Enterocystoplasty is associated with a low but distinct risk of malignancy (Greenwell *et al*, 2001; Ali-El-Dein *et al*, 2002) and cancers arising within enterocystoplasties are aggressive and associated with an extremely poor prognosis (Filmer and Spencer, 1990). Studies using CGH identified chromosomal abnormalities suggestive of genetic instability at the enterovesical anastomosis of patients with an enterocystoplasty, which were not present in the native bladder remnant (Appanna *et al*, 2000; Appanna, 2004). The results of the present study are in accord with these observations (Appanna *et al*, 2000; Appanna, 2004) and clearly indicate that genetic changes are present at the enterovesical anastomosis of patients who have undergone a clam ileocystoplasty prior to the development of any histological change. In particular, aneusomy of chromosome 18 was present in 87% (13 out of 15) of patients, indicating that there may be genes on this chromosome that confer a particular selective growth advantage within the anastomosis microenvironment. It is not possible from the results of the present study to determine whether the chromosomal changes observed with FISH are essential to the development of tumorigenesis in a clam enterocystoplasty. However, genomic instability is believed to be essential for tumorigenesis, rendering the cancer cell genome more susceptible than normal cells to the development of the various abnormalities characteristic of neoplasia (Marx, 2002).

In the present study, there was no correlation between the number of aneusomic changes and the time between a patient undergoing operation and subsequent biopsy, partly because the cohort studied was not large enough and partly because the time between operation and biopsy (range 6–18 years) was not great enough to detect such associations. It is also possible that local factors are different for each patient and that the process of tumorigenesis occurs at a different rate in individual patients, a suggestion supported by the very wide range (2–40 years) of reported latency between operation and presentation of tumour (Lane and Shah, 2000; Sato *et al*, 2000). Future studies need to be directed at defining the temporal relations between operation and the development of chromosomal alterations at the enterovesical anastomosis in patients who have undergone enterocystoplasty.

Cancers forming within enterocystoplasties are extremely aggressive, with a high attendant mortality (Filmer and Spencer, 1990). In 1990 a review identified 14 cases of carcinoma formation within an enterocystoplasty (Filmer and Spencer, 1990), and since that time the number of reported cases has more than trebled. This paper provides the 48th description of a malignant tumour within an augmentation cystoplasty reported in the literature. In this study, the patient presented between-yearly screening with an advanced tumour and died despite radical surgery. Moreover, five of the six patients who have presented with a clam tumour at our own unit have died. All of the tumours presented incidentally and none were detected by screening, suggesting that currently accepted yearly cystoscopic surveillance is inadequate and emphasising the urgent need to develop suitable methods for identifying those patients at risk of tumour formation early in the process of tumorigenesis.

The most striking cytogenetic finding in the clam tumour was the large number of polysomic cells. Polysomy is known to be an unfavourable prognostic marker and is associated with rapid progression of a cancer (Gschwendtner and Mairinger, 1999; Krause *et al*, 2003; Deliveliotis *et al*, 2005). Therefore, the presence of polysomy within this fatal squamous cell carcinoma was consistent with the aggressive nature of this tumour. Further studies are required to establish whether such complex chromosomal abnormalities are a typical feature of clam cancers. Generally, where polysomic cells are reported as a feature of human cancer, they typically have three or four times the haploid number of chromosomes present. Very occasionally, cells with higher multiples of the haploid chromosome number have been reported and are associated with a poor prognosis (Borgström *et al*, 1976). In the present study, the majority of the tumour polysomic cells were either trisomic or tetrasomic. However, about 10% of the polysomic cells displayed gross abnormalities and showed as many as 16 signals for a given probe, suggesting that, in the squamous cell carcinoma reported here, there was a severe disturbance that was reflected in the clinical outcome. The findings are therefore in accord with a previous study, which indicated that hypertetraploidy was the most important risk factor for tumour progression and clinical outcome of patients with superficial bladder cancer (Tachibana *et al*, 1999).

It seems unlikely that every patient with an enterocystoplasty is at a high risk of developing a life-threatening tumour as the clam tumours described in the literature have presented with a highly variable latency. Therefore, the finding of generalised chromosomal changes appears to be a nonspecific marker of cytogenetic instability and may not be helpful in identifying those patients at a high risk of cancer formation. In the present study, aneusomy of chromosome 18 appeared to be a good marker for genomic instability at the enterovesical anastomosis, but concomitant aneusomy of chromosomes 8 or 9 may be a more discerning marker to identify those patients who will develop a cancer. Polysomy was not identified in any of the surveillance biopsies taken from the enterovesical anastomosis, but was the most prevalent finding in the squamous cell clam tumour analysed using FISH. If polysomy is demonstrated at the enterovesical anastomosis following a clam enterocystoplasty in any patients in future, they should be carefully followed up.

## CONCLUSIONS

In conclusion, since cancers occurring within clam ileocystoplasties are highly aggressive and have developed rapidly by the time they are diagnosed, a method that predicts tumour formation would be of obvious benefit in the management of these patients. The results of the present study indicate that chromosomal losses and gains suggestive of genetic instability are present in morphologically normal tissue obtained from patients with an enterocystoplasty. However, further prospective, longitudinal, blinded studies are required to establish whether or not predetermined abnormal FISH signal patterns predict the presence or absence of tumour. Several chromosomal changes were observed in this study. In particular, chromosome 18 aneusomy was prevalent and may be a useful marker of genetic instability. Identification of polysomic cells at the enterovesical anastomosis of patients who have undergone an enterocystoplasty would be a cause for concern. However, longitudinal follow-up of patients is required to confirm whether a particular type of cytogenetic abnormality is associated with tumour formation.

## Figures and Tables

**Table 1:**
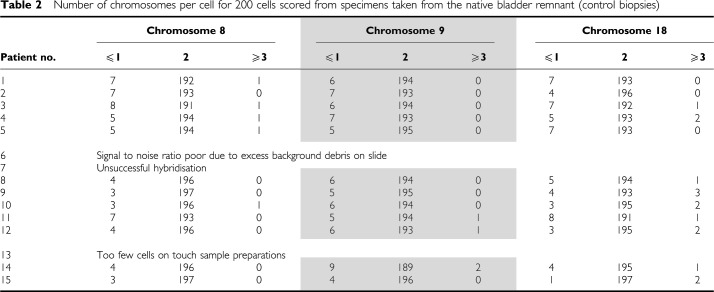
Number of chromosomes per cell for 200 cells scored from specimens taken from the enterovesical anastomosis

**Table 2:**
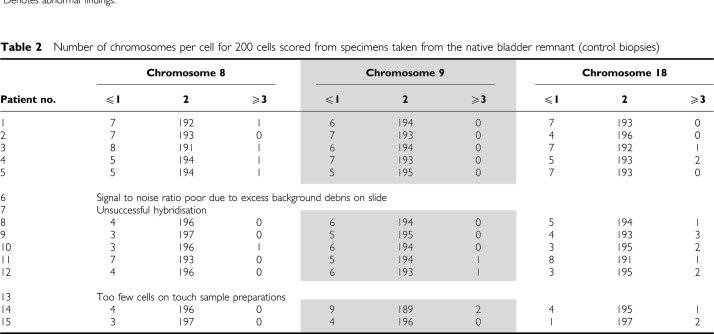
Number of chromosomes per cell for 200 cells scored from specimens taken from the native bladder remnant (control biopsies)

**Table 3:**
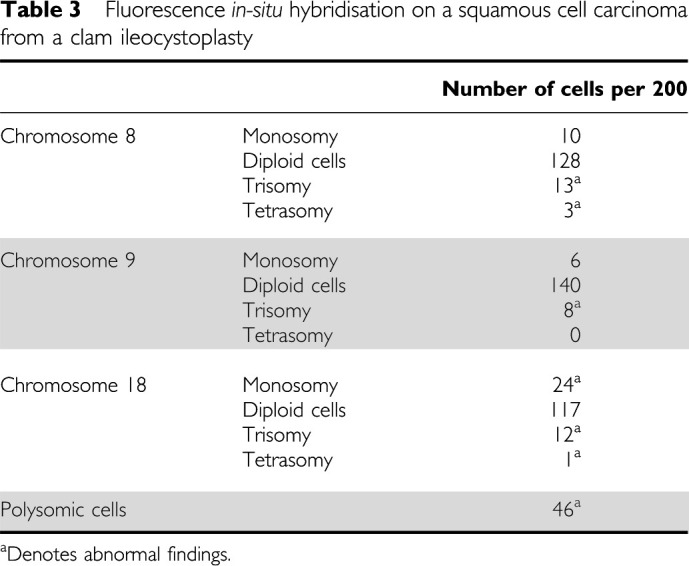
Fluorescence *in-situ* hybridisation on a squamous cell carcinoma from a clam ileocystoplasty
